# Deconvoluting the Complexity of Reactive Oxygen Species (ROS) in Neurodegenerative Diseases

**DOI:** 10.3389/fnana.2022.910427

**Published:** 2022-06-09

**Authors:** Yuxin Zhou, Yongqi Zhen, Guan Wang, Bo Liu

**Affiliations:** State Key Laboratory of Biotherapy and Cancer Center, West China Hospital, Sichuan University, Chengdu, China

**Keywords:** reactive oxygen species (ROS), oxidative stress, Alzheimer’s disease, Parkinson’s disease, amyotrophic lateral sclerosis

## Abstract

Neurodegenerative diseases (NDs) are becoming a serious public health concern as the world’s population continues to age, demanding the discovery of more effective therapies. Excessive formation of reactive oxygen species (ROS) can result in oxidative stress (OS), which can be regarded as one of the common causes of neurodegenerative diseases (NDs). Thus, in this review, we focus on summarizing the consequences of ROS NDs, while taking the four prevalent NDs as examples, including Alzheimer’s disease (AD), Parkinson’s disease (PD), Amyotrophic lateral sclerosis (ALS), and Huntington’s disease (HD), to illustrate the key signaling pathways and relevant drugs. Together, these findings may shed new light on a field in which ROS-related pathways play a key role; thereby setting the groundwork for the future therapeutic development of neurodegenerative diseases.

## Introduction

**Figure 1:**
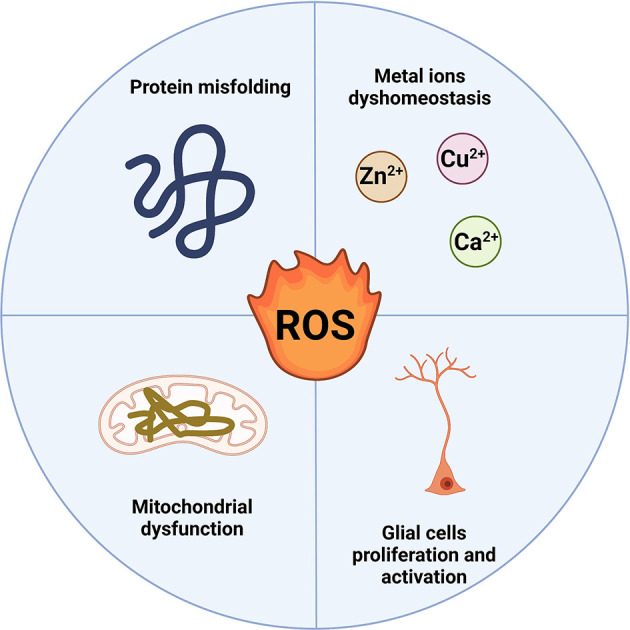
ROSproduction in neurodegenerative diseases. Protein misfolding, metal ions dyshomeostasis, mitochondrial dysfunction, and glial cell proliferation and activation mainly induce the ROS production in NDs.At the same time, the overproduction of ROS can also affect the four pathological processes (Created with BioRender.com). ROS, reactive oxygen species; NDs, neurodegenerative diseases.

Neurodegenerative diseases (NDs) are a diverse set of illnesses characterized by the slow loss of anatomically or physiologically relevant neural systems. They are common causes of morbidity and cognitive impairment in the elderly, including Alzheimer’s disease (AD), Parkinson’s disease (PD), Amyotrophic lateral sclerosis (ALS), Huntington’s disease (HD), etc. (Erkkinen et al., 2018). Amyloidoses, tauopathies, α-synucleinopathies, and TDP-43 proteinopathies are the most frequent conditions in which proteins exhibit aberrant structural features. Excessive production of reactive oxygen species (ROS) has been reported to play an important role in these protein misfolds (Poprac et al., 2017). The main sources of cellular ROS are mitochondria and NADPH oxidases (NOXs). In most cells, the mitochondrial electron transport chain (ETC) is one of the most important sources of reactive oxygen species (ROS), with a research reporting that mitochondria generate 45% of ROS while NOXs account for the remaining 40% (Wong et al., 2019). Under physiological settings, the balance between the production of ROS and the clearance of ROS is extremely tightly controlled. When the delicate equilibrium is disturbed in some pathogenic conditions, including mitochondrial dysfunction, protein misfolding, metal ions dyshomeostasis, and glial cells proliferation and activation (Yeung et al., 2021), ROS levels rise, resulting in OS which contributes significantly to the degeneration of neuronal cells by interfering with the function of biomolecules (DNA, protein, and lipid; Figure 1). As a result, ROS involved in neurodegenerative changes has become a research hotspot.

Thus, ROS regulation has emerged as a promising strategy in the NDs field. So far, none of the FDA-approved small molecule drugs for NDs therapies have a clear mechanism for targeting ROS, and there is an urgent need to figure out the complexity of ROS in NDs and identify potential targets in the ROS-related pathway for therapy requirements. Herein, we focus on summarizing the ROS-regulated molecular mechanisms in NDs and their relevant molecular drugs over the recent 5 years.

## ROS in Neurodegenerative Diseases

**Figure 2:**
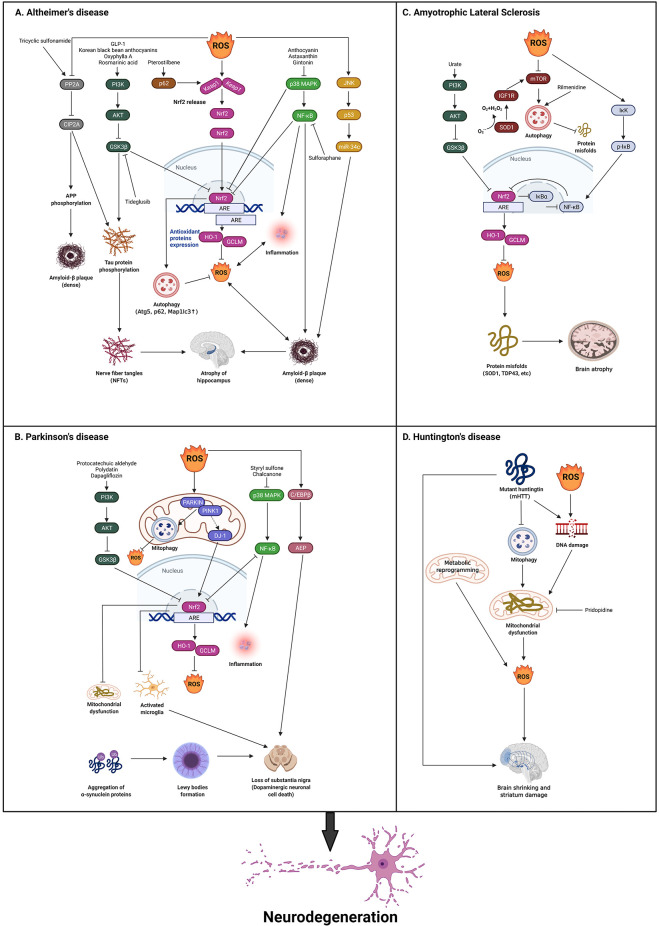
Theroles of reactive oxygen species (ROS) in neurodegenerative diseases.The presence of hallmark protein(s) for each neurodegenerativedisease is a common trait, such as Tau and Aβ in AD,α-synuclein in PD, TDP-43, and SOD1 in ALS, and mHTT in HD.**(A)** In AD, ROS production serves both as a stimulus and aconsequence of activated Nrf2 *via* PI3K/AKT/GSK3β,p62, p38 MAPK/NF-κB pathways, which is demonstrated toclosely correlates with AD pathogenesis. Besides, ROS induced ADdevelopment through the inhibition of PP2A/CIP2A and the activation of JNK/P53 pathways. Some corresponding drugs are utilized to reverse this and exhibit some initial effects. **(B)** In PD, ROS also acts as both stimuli and a consequence of activated Nrf2 *via* PI3K/AKT/GSK3β, DJ-1, and p38 MAPK/NF-κB pathways. Additionally, ROS can activate c/EBPβ/AEP pathway, which leads to dopaminergic neuronal loss and motor disorders. Some drugs are found to reverse the pathology through the above-mentioned pathways. **(C)** In ALS, the inhibition of GSK3β is reported to activate Nrf2 *via* PI3K/AKT pathway. SOD1 is an important gene that is relevant to ROS in ALS that inhibits ROS production. ROS activates IκK/p-IκB/NF-κB pathway to inactivate Nrf2. The activation of SOD1 also regulates IGF1R/mTOR pathway to inhibit autophagy which can eliminate misfolded proteins. Rilmenidine is found to reduce autophagy to alleviate ALS development. **(D)** In HD, mHTT blocks autophagy, and mHTT and the over production of ROS leads to DNA damage to produce ROS through mitochondrial dysfunction, which results in ALS. Metabolic reprogramming can also induce ROS production which leads to ALS. Finally, pridopidine can be found to inhibit mitochondrial dysfunction to reverse this pathology (Created with BioRender.com). AD, Alzheimer’s disease; PD, Parkinson’s disease; HD, Huntington’s disease; ALS, amyotrophic lateral sclerosis; SOD1, Superoxide Dismutase 1; TDP-43, TAR DNA-binding protein 43; Nrf2, Nuclear factor erythroid 2-related factor 2.

Occurring as one of the primary hallmarks of a variety of clinical conditions, oxidative stress (OS) is produced by the unchecked generation of ROS, which promotes severe damage to brain tissue. The functions of ROS in the development of NDs are still unclear. In this review, we describe four categories of common NDs and the potential impacts of ROS in these NDs (Figure 2).

## Alzheimer’s Disease

AD is one of the most common NDs, impacting 45 million individuals worldwide. Deposition of protein aggregates, including extracellular amyloid plaques (Aβ), intracellular tau (forms nerve fiber tangles), and loss of synaptic connections in specific areas of the brain characterize AD (Knopman et al., 2021). It has been reported that in the early stage of AD, oxidative damage occurs in the brain before significant plaque pathology develops (Butterfield and Halliwell, 2019).

Several pathways connecting ROS in AD have recently been uncovered. Nuclear factor erythroid 2-related factor 2 (Nrf2) is a crucial redox-regulated gene in controlling ROS levels, with intranuclear Nrf2 decreased in NDs such as AD (Cores et al., 2020). Kelch-like ECH-associated protein 1 (Keap1) and antioxidant response element (ARE) are important to Nrf2 pathway. Keap1-Nrf2-ARE can be divided into two parts: the cytoplasm and the nucleus. Under normal circumstances, Keap1 binds with Nrf2 in the cytoplasm and stays in an inactive state, where Nrf2 will be ubiquitinated and then degraded. When stimulated by ROS, the binding of Keap1-Nrf2 is unstable. Nrf2 is released and transferred to the nucleus then binds to ARE and promotes the transcription of downstream genes, leading to the translation of a series of related proteins to exert physiological effects (Osama et al., 2020). These proteins include heme oxygenase-1 (HO-1), glutathione cysteine ligase modulatory subunits (GCLM), etc. which are antioxidant proteins that can reduce ROS production. Nrf2 can also promote autophagy, which helps remove Aβ aggregates and phosphorylated Tau proteins. When Nrf2 binds ARE, the transcription of autophagy-related genes like Atg5, p62, and Map1lc3b are also upregulated. The inhibition of Nrf2 pathway and the dysfunction of autophagy will in turn cause the accumulation of ROS, senescent organelles, and misfolded proteins (Zhang W. et al., 2021).

A growing number of studies have proved that the activation of nuclear Nrf2 is affected by phosphatidylinositol 3-kinase (PI3K), Akt, and GSK3β. PI3K is a dimer composed of the regulating subunit p85 and the catalytic subunit p110. When it binds to growth factor receptors, it can alter and activate the Akt protein structure and activate or inhibit a series of substrates downstream by phosphorylation (Vidal et al., 2021), including the inhibition of GSK3β *via* phosphorylation at Ser9. GSK3β can phosphorylate Nrf2, causing the Nrf2 nuclear export and degradation (Fão et al., 2019). As studies have shown elevated GSK3β levels in AD and increased GSK3β activity are directly involved in the degradation of Nrf2, the inhibition of GSK3β may be a possible therapeutic strategy for the treatment of AD. In AD mice, a GSK3β inhibitor called tideglusib can reduce tau phosphorylation, decrease Aβ deposition, and increase astrocyte proliferation (Lauretti et al., 2020). GLP-1 has been shown to improve AD cognition by alleviating Aβ-induced glycolysis declines in astrocytes to reduce ROS production *via* PI3K/Akt pathway (Zheng et al., 2021). Korean black bean anthocyanins, a natural antioxidant neuroprotective compound, reduced synaptic and memory loss and neurodegeneration in an AD model by inhibiting Aβ-induced ROS-mediated OS *via* the PI3K/Akt/GSK3β/Nrf2 pathway *in vitro* and *in vivo* (Ali et al., 2018). Oxyphylla A, a compound extracted from Alpinia oxyphylla, has been also found to reduce Aβ proteins in SAMP8 mice *via* the activation of the Akt/GSK3β pathway to activate Nrf2 and reduce ROS (Bian et al., 2021). Rosmarinic acid (RosA) shows the same effect as Oxyphylla A, and it has been reported to attenuate Aβ-induced cellular ROS generation in PC12 cells (Rong et al., 2018).

Meanwhile, p62 is an intracellular signaling protein involved in a variety of cellular environments. Several reports have proved that p62 can promote Nrf2 activity by triggering Keap1 (Sánchez-Martín et al., 2020). The phosphorylation of p62 at ser349 strongly enhances its interaction with Keap1, which results in Nrf2 dissociation and activation (Ichimura and Komatsu, 2018). Pterostilbene has been reported to activate the Nrf2 pathway by promoting the binding of p62 and Keap1 in SH-SY5Y cells, which results in the downregulation of ROS (Xu et al., 2021a). It has been reported that the activation of the Nrf2-mediated p62 signaling pathway can induce autophagy to reduce the Aβ caused cell death in PC12 cells. Autophagy inhibited ROS generation by facilitating mitochondrial turnover as well (Gu et al., 2018). The p62 also plays an effective and specific role in the clearance of microtubule-associated protein tau (MAPT) by blocking nerve fiber tangles accumulation and pathological diffusion (Xu et al., 2019).

Besides, the p38 mitogen-activated protein kinase (MAPK), which was found upregulated in AD, has been proved to reduce the nuclear transfer of Nrf2. Anthocyanin, a subfamily of flavonoids with antioxidation, has been reported to reduce ROS expression through increased Nrf2 and HO-1 protein levels in SH-SY5Y cells *via* inhibiting p38 MAPK (Amin et al., 2017). Astaxanthin has also been found to reduce ROS and neuronal death through the p38 MAPK signaling pathway (Zhang X. S. et al., 2021).

Furthermore, the transcription factor nuclear factor-κB (NF-κB) is regulated in a complex manner. It is a master switch of inflammation that is associated with H_2_O_2_ production and is also related to Nrf2 regulation (Sies and Jones, 2020). Gintonin, a glycolipoprotein fraction isolated from ginseng, has been reported to inhibit p38 MAPK and NF-κB pathways to stabilize Nrf2 to reduce ROS (Choi et al., 2021). Sulforaphane, another positive modulator of Nrf2, reduces Aβ and ROS *via* the inhibition of NF-κB. It also decreases pro-inflammatory cytokine expression and p65 activation, resulting in increased protein expression levels of HO-1 (Zhao et al., 2018).

Apart from the Nrf2 pathway, other mechanisms have been found in the regulation of ROS in AD. For instance, protein phosphatase 2A (PP2A), a ubiquitously expressed serine/threonine phosphatase can be inhibited by ROS. PP2A has been proved to inhibit CIP2A in order to phosphorylate Tau and amyloid precursor protein (APP) in mouse brains. Synthetic tricyclic sulfonamide PP2A activators have been proven to decrease Tau and APP phosphorylation *via* this pathway (Wei et al., 2020). Moreover, microRNAs (miRNAs) are small, endogenous, non-coding RNAs that act as regulators in a variety of biological processes. The expression changes in miRNAs may cause diseases. The miR-34c has been found upregulated in AD, with intracellular Aβ aggregation and tau hyperphosphorylation in different regions of the brain, together contributing to cognitive deficits (Bazrgar et al., 2021). A recent study shows that the upregulated miR-34c participates in the pathogenesis of AD *via* ROS/JNK/P53 pathway and the inhibition of miR-34c can improve memory decline in AD models (Shi et al., 2020).

## Parkinson’s Disease

PD is the second most common ND, with a prevalence of more than 6 million worldwide. Neuronal loss in the substantia nigra (SN) is a neuropathological characteristic of PD, which leads to striatal dopaminergic insufficiency and the buildup of α-synuclein in neuronal inclusions. The α-synuclein binds to ubiquitin and forms proteinaceous cytoplasmic inclusions of proteins called Lewy bodies (Zhang K. et al., 2021). Disturbance of physical process and pathway dysfunction, including OS, defective mitochondria, and cellular calcium imbalances, plays a part in ROS imbalance, which is consequently involved in PD etiology all play a part in the etiology of PD (Aarsland et al., 2021). Furthermore, due to lower glutathione (GSH) levels, the inherent antioxidant defenses in dopaminergic neurons in SN pars compacta are more vulnerable to ROS than in other parts of the brain (Bjørklund et al., 2021).

Nrf2 is also the main protein involved in the development of ROS-caused PD, while the Akt/GSK3β/Nrf2 axis is extensively targeted by drugs. Protocatechuic aldehyde (PCA) has been found to perform an effective neuroprotective role in MPTP or MPP^+^ generated PD mice (Guo et al., 2019) by correcting mitochondrial dysfunction and relieving ROS damage *via* the GSK3β/Nrf2 pathway. In addition, schisandra chinensis (Sch) was reported to reduce GSK3β activity and upregulate Nrf2 in the striatum and hippocampus, block NF-κB nuclear translocation, and ameliorate excessive ROS levels in a 6-OHDA-induced PD model (Yan et al., 2021). Polydatin has also been reported to prevent dopaminergic neurodegeneration by inhibiting microglia activation through AKT/GSK3β/Nrf2 signaling pathway in lipopolysaccharide (LPS)-induced PD models (Huang et al., 2018). Moreover, dapagliflozin reduces ROS production in the rotenone-induced PD model *via* the activation of the PI3K/AKT/GSK3β pathway, which results in the attenuation of neuronal injury (Arab et al., 2021). The p38 MAPK and NF-κB have also been researched in PD. Overproduction of ROS results in the activation of MAPK and NF-κB pathways, providing links between OS and neuroinflammation. A novel synthetic styryl sulfone and a novel chalcone compound have been published to prove this statement. These compounds activate Nrf2 to produce HO-1, which inhibits the production of ROS and the p38 MAPK and NF-κB mediated neuroinflammation and in PD models, rescues the dopamine neurotoxicity (Lee et al., 2019; Guo et al., 2021).

Besides those similar pathways in AD pathology, some extra pathways to affect ROS are found in PD. For example, excessive ROS production can diminish mitochondrial membrane potential (MMP), causing the accumulation of PTEN induced putative kinase 1 (PINK1) and E3 ubiquitin ligase Park 2 (Parkin), which activates mitophagy to reduce ROS by combining sequestosome 1 (p62) and microtubule-associated protein 1 light-chain 3 (LC3; Cui et al., 2021). The mutation of PINK1 and Parkin may block mitophagy, leading to the accumulation of defective mitochondria, ROS increases, and ultimately to neurodegeneration (Wen et al., 2013). In the rotenone-induced PD model, the rotenone treatment has been found to activate p38 MAPK which disrupts mitophagy and results in ROS increase. And the ROS inhibitor NAC provided protection by restoring cell death and mitochondrial function in this model (Chen et al., 2021). In addition to functional interactions with PARKIN, PINK1 can also fight ROS by interacting with DJ-1 as a neuroprotective protein. DJ-1 is a small 20 kDa protein that is highly conserved in different species. It can be oxidized at its cysteine residue under OS, thus acting as a ROS scavenger. DJ-1 also stabilizes Nrf2 to enhance antioxidant response (Zhao et al., 2021). Interestingly, another study has reported that MEHP upregulated ROS production to activate mitophagy, which increases cytotoxicity as a mechanism of cell death (Xu et al., 2021b). Therefore, ROS should be further studied in the mitophagy-related pathogenesis PD pathogenesis.

Recently, scientists identified TTFA (a complex II inhibitor) and Atovaquone (a complex III inhibitor), which are effective in blocking oxidative phosphorylation, strongly elevating ROS, and activating dopaminergic neuronal cell death through the C/EBPβ/AEP pathway, leading to PD (Ahn et al., 2021).

## Amyotrophic Lateral Sclerosis

ALS is a progressive, fatal neuromuscular disorder characterized by the degeneration of upper and lower motor neurons leading to somatic muscle dysfunction in the body (Grad et al., 2017). According to the ALS Association, ALS affects roughly 1 in 50,000 people worldwide each year. ALS is implicated in a range of pathogenesis, such as excitatory toxicity, mitochondrial dysfunction/dysregulation, endoplasmic reticulum stress, neuroinflammation, and OS (D’Ambrosi et al., 2018). The loss of nuclear TAR DNA-binding protein 43 (TDP-43) function may contribute to the progression of ALS (Tziortzouda et al., 2021). Importantly, increased ROS has been implicated in the etiology of ALS in a number of studies. ROS markers rise in the postmortem brains of people with ALS, as well as in transgenic animal models (D’Ambrosi et al., 2018).

In the majority of ALS cases, which are defined as sporadic (SALS), the etiology of the disease is unknown, while 5%–10% of cases are hereditary and categorized as familial (FALS). Chromosome 9 Open Reading Frame 72 (c9orf72), Superoxide Dismutase 1 (SOD1), TDP-43, Fused in Sarcoma (FUS), Optineurin (OPTN), and TANK-binding kinase 1 (TBK1) are among the FALS-related genes. SOD1, is the first recognized gene linked to ALS, whose mutations account for roughly 20% of familial forms of ALS (McCampbell et al., 2018). Functional SOD1 encodes a Cu^2+^/Zn^2+^-binding SOD, and converse O_2_^•−^ to H_2_O_2_ and O_2_ to protect cells from toxic ROS, while in SOD^G93A^ mice, a classic ALS model, ROS levels increase due to the SOD1 mutation (Xiao et al., 2018).

Similarly, the Keap1/Nrf2 complex is important in controlling ROS levels in ALS, much as it is in AD and PD. Kirby and colleagues found the first indication of a link between SOD1 and Nrf2 when mut-SOD1 (G93A) reduced Nrf2 mRNA expression in the mouse motor neuron-like hybrid cell line NSC34. The mutant SOD1 models also demonstrate ARE dysfunction which leads to ROS overproduction (Kirby et al., 2005). As written above, GSK3β and NF-κB are also key factors in ALS. Urate has been proved to decrease ROS *via* Akt/GSK3β/Nrf2/GCLC pathway to protect motor neurons in the ALS model (Zhang et al., 2019). Neuronal-specific inhibition of IκB lowers motor neuron loss and reactive glial cells in SOD1^G93A^ mice and TDP-43 mice by reducing misfolded SOD1 levels and TDP-43 translocation into the nucleus. These findings improved the cognitive impairment of ALS transgenic mice, allowing longer lifespans (Dutta et al., 2020). In addition, inhibiting NF-κB in microglia and astrocytes can reduce brain and peripheral inflammation, as well as extend mouse survival (Ibarburu et al., 2021). Under ROS, Nrf2/ARE signaling is a critical protective strategy for cell survival. ROS stimulates IκB kinase (IκK) activation and then mediates IB (NF-κB inhibitor) phosphorylation, increasing proteasome degradation and NF-κB release. However, at the transcriptional level, the two opposing pathways can interfere with each other. Nrf2 reduces ROS-mediated NF-κB activation by boosting ROS-neutralizing antioxidant defenses, which decreases NF-κB pathway activation (Sivandzade et al., 2019).

Additionally, autophagy is the principal intracellular catabolic route for removing misfolded proteins, aggregates, and damaged organelles that cause aging and neurodegeneration like ALS, in which autophagy is frequently disrupted, leading to cytoplasmic separation of the readily aggregated and toxic proteins in neurons, especially dysfunctional SOD1 to produce ROS. Since autophagy is regulated through mTOR-dependent and -independent mechanisms, mTOR is considered as a key target to rescue the impaired autophagy. Accordingly, increasing levels of ROS were found to cause the reduction of mTOR in the larval brain (Chaplot et al., 2019). And the stimulation of the mTOR system in mutant SOD1 astrocytes is caused by post-transcriptional overexpression of IGF1R (insulin-like growth factor 1 receptor), an upstream positive modulator of the mTOR pathway, according to a recent study. Astrocytes with mutant SOD1 are less toxic to motor neurons when the IGF1R-mTOR pathway is inhibited (Granatiero et al., 2021). Rilmenidine has been reported to induce autophagy in mutant SOD1^G93A^ mice, which results in the downregulation of SOD1 and ROS reduction (Perera et al., 2018).

## Huntington’s Disease

A genetic ailment characterized by movement abnormalities and cognitive deterioration, Huntington’s disease (HD) is inherited in an autosomal dominant manner. Symptoms of HD include a general shrinking of the brain and degeneration of the striatum (caudate nucleus and putamen), as well as the loss of efferent medium spiny neurons in the striatum (caudate nucleus and putamen; MSNs). These symptoms may be related to the widespread expression of mutant huntingtin (the toxic protein that causes HD) in HD patients’ bodies (Jimenez-Sanchez et al., 2017).

ROS and mitochondrial dysfunction have a role in the neuronal degeneration of HD. A genome-wide association study (GWAS) containing 6,000–9,000 patients identifies DNA repair related genes as major modulators of age at onset and disease severity, with some pathways connected to redox signaling and mitochondrial function. In the presence of ROS, huntingtin works as a scaffold that can localize to DNA damage and modifies its associated complex (Maiuri et al., 2019). Other research demonstrates that huntingtin is engaged in a variety of mitophagy processes. The existence of the polyglutamine tract in mutant huntingtin alters the formation of these protein complexes and determines mutant huntingtin’s deleterious effects on mitophagy, which result in a buildup of damaged mitochondria and an increase in ROS. In HD, these alterations lead to overall mitochondrial dysfunction and neurodegeneration (Franco-Iborra et al., 2021). Pridopidine has been reported to increase mitochondrial respiration and reduce ROS in HD models (Naia et al., 2021). ROS has also been linked to Huntington’s disease-associated region-specific cell death. Studies have demonstrated that mitochondria can undergo metabolic reprogramming by utilizing fatty acids as a source of energy, causing ROS-induced damage in the vulnerable striatum (Polyzos et al., 2019).

## Conclusions

In this review, we provided an illustration of the responsibilities of ROS in NDs and summarized some related drugs for potential therapeutic purposes. ROS is primarily produced by mitochondria and NOXs, which can cause OS. Some of the intricate mechanisms in which ROS can contribute to the development of NDs have been elucidated, with Nrf2 as the central regulator of ROS in NDs, while other cellular processes such as mitophagy, and neuroinflammation play their crucial roles in controlling ROS in NDs. For instance, in AD, Nrf2 and its related pathways are widely reported. In PD, the influence on mitophagy PINK1/PARKIN pathway has also been further discussed. In ALS, the relationship between SOD1 and ROS has been illustrated, and in HD, ROS has been suggested to affect metabolic reprogramming. However, most of these studies, especially those related to HD, lack in-depth investigations on the specific role of ROS in NDs, when only covering the measurement of the alterations of ROS, which can be a consequence or concomitant phenotype in response to NDs. Thus, more detailed relationships between ROS and the occurrences of NDs should be further exploited.

Moreover, in the concept of alleviating OS caused by ROS, some nutritional factors (e.g., resveratrol and curcumin) that act as antioxidants have been guided to treat NDs. But the use of these antioxidants to control and prevent NDs remains unsatisfactory. The reason for this is that most antioxidants have osmotic limitations due to their inability to pass the blood-brain barrier. Nanoparticles may prove to be an effective vehicle for delivering these medications to the central nervous system with the advancement of nanotechnology. We propose that, rather than using scavengers, direct regulation of ROS production from specific sources with targeted drugs should be used to avoid or limit oxidative damage in neurodegeneration. It has been proven that several ND-related genes/proteins like Nrf2, GSK3β, p38 MAPK, etc., are involved in the regulation of the ROS pathway. By focusing on specific ROS-mediated signaling pathways, we can anticipate the development of more refined redox drugs. Direct inhibition of an enzyme, increased endogenous antioxidants, or increased energy production, will be a promising direction for future therapeutic purposes in NDs.

## Author Contributions

YZho and YZhe wrote the manuscript. GW and BL designed and supervised this article. All authors contributed to the article and approved the submitted version.

## Conflict of Interest

The authors declare that the research was conducted in the absence of any commercial or financial relationships that could be construed as a potential conflict of interest.

## Publisher’s Note

All claims expressed in this article are solely those of the authors and do not necessarily represent those of their affiliated organizations, or those of the publisher, the editors and the reviewers. Any product that may be evaluated in this article, or claim that may be made by its manufacturer, is not guaranteed or endorsed by the publisher.
